# Danazol in Refractory Autoimmune Hemolytic Anemia or Immune Thrombocytopenia: A Case Series Report and Literature Review

**DOI:** 10.3390/ph15111377

**Published:** 2022-11-09

**Authors:** Hsu-En Huang, Ko-Ming Lin, Jing-Chi Lin, Yu-Ting Lin, Hsiao-Ru He, Yu-Wei Wang, Shan-Fu Yu, Jia-Feng Chen, Tien-Tsai Cheng

**Affiliations:** 1 Division of Rheumatology, Allergy and Immunology, Department of Internal Medicine, Chiayi Chang Gung Memorial Hospital, Chiayi 613, Taiwan; 2 Division of Rheumatology, Allergy and Immunology, Department of Internal Medicine, Kaohsiung Chang Gung Memorial Hospital, Kaohsiung 833, Taiwan; 3 School of Medicine, College of Medicine, Chang Gung University, Taoyuan 333, Taiwan

**Keywords:** danazol, autoimmune hemolytic anemia, immune thrombocytopenia

## Abstract

Danazol is a treatment option for autoimmune hemolytic anemia (AIHA) and immune thrombocytopenia (ITP). Three patients with AIHA and eight patients with ITP between 2008 and 2022 were enrolled in the Rheumatology Outpatient Clinic of Chang Gung Memorial Hospital, Kaohsiung. Those patients were refractory or intolerant to conventional therapy and were treated with danazol. All the patients received an initial dose of danazol (200–400 mg). The observation period was 6 months. Three patients (100%) with AIHA and six (75%) with ITP achieved treatment response after 6 months of danazol therapy. The dose of glucocorticoid for responders could be reduced to ≤5 mg/day of prednisolone, and the immunosuppressants, except hydroxychloroquine and azathioprine for systemic lupus erythematosus, could be discontinued. Adverse events were acne in two (18.2%) patients and transient dose-related liver function impairment in one (9.1%) patient in the current series. Danazol therapy appears to be a favorable alternative for refractory AIHA and ITP by altering the erythrocyte membrane to resist osmotic lysis and protecting platelets against complement-mediated lysis. In this report, we also performed a literature review and searched the PubMed/Cochrane Library for articles published from 1984 to January 2022 on danazol therapy for patients with AIHA and ITP.

## 1. Introduction

Autoimmune hemolytic anemia (AIHA) and immune thrombocytopenia (ITP) are two common immune-mediated hematologic diseases that are usually initially managed with high-dose glucocorticoids (GCs) [1], cytotoxic agents, or immunosuppressants, such as azathioprine (AZA), cyclophosphamide (CYC), cyclosporine (CsA), and mycophenolate mofetil (MMF). Despite the availability of various regimens, the best response rate to glucocorticoid (GC)-based combination therapy for these diseases is 50–91% [2,3].

However, 60–80% of patients with these diseases experience recurrence after tapering the dose of GCs [4,5]. Therefore, approximately 20% of patients need long-term maintenance therapy or splenectomy to prevent a flare-up of the disease. Long-term therapy with the aforementioned medications or splenectomy is associated with several sequelae such as osteoporosis, cytopenia, and immune-compromised associated infections.

In addition to conventional immunosuppressants, several novel therapies have been developed for refractory AIHA or ITP. Rituximab (RTX) is the most widely used biologics in immune-mediated hematologic diseases and has a response rate of around 75% and 58–81% in AIHA [6,7] or ITP [8,9], respectively. However, in the context of the COVID-19 pandemic era, RTX treatment might affect the antibody response to SARS-CoV-2 vaccination [10], and it seems RTX is inadequate for patients with active AIHA and ITP under high-dose GC therapy. Other medications with different mechanisms of action, including a proteasome inhibitor (bortezomib) and thrombopoietin-receptor agonists (eltrombopag, romiplostim, and avatrombopag) appear effective and well-tolerated in multi-relapsed AIHA [11] or refractory ITP [12,13,14], respectively. However, these medications are subjected to very high costs and lead to potentially adverse events [15,16].

Danazol is a weak androgen and anabolic steroid and is indicated in relation to endometriosis. Several case series reports have illustrated the effect of higher doses of danazol (400–800 mg/day) on AIHA, ITP, and danazol-related adverse events (AEs) in treating these diseases.

This study aimed to share the experience of using danazol with lower initial and maintenance doses to treat patients with AIHA or ITP who were refractory or intolerant to conventional medications or surgical intervention. In addition, in this report, we reviewed the literature pertaining to the effect of danazol on AIHA or ITP.

## 2. Results

### 2.1. Case Series

Eleven patients were enrolled, including three (27%) with AIHA and eight (73%) with ITP. Demographics, clinical characteristics, treatment responses, and AEs are summarized in Table 1. All the participants were female. The age of the patients who received danazol was 52.6 ± 10.2 years. The disease duration at the initiation of danazol was 13.5 ± 8.6 years. The leading concomitant disease was systemic lupus erythematosus (SLE) in nine patients (82%). The observation period was 6 months for response in the current series. The time of response to danazol was 2.0 ± 1.1 (range, 1 to 4) months. The initial daily dose of danazol was 200 mg in four (36%) patients and 400 mg in seven (64%) patients.

**Figure 1:**
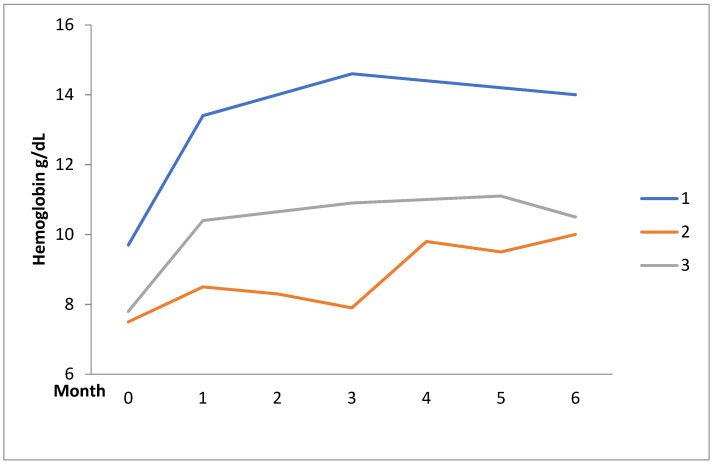
Serial changes in hemoglobin levels over time in autoimmune hemolytic anemia after initiation of danazol.

**Figure 2:**
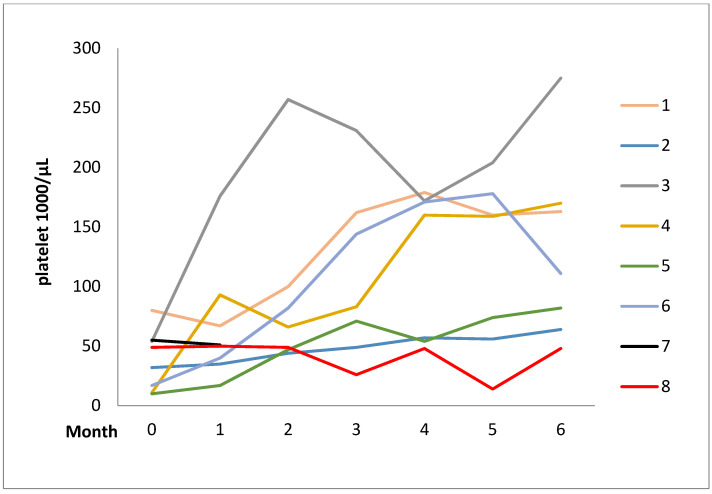
Serial changes in platelet levels over time in immune thrombocytopenia after the initiation of danazol.

All the patients received moderate-to-high-dose GC treatment (prednisolone 0.5–1 mg/kg/day or equivalent) and immunosuppressant agents, including hydroxychloroquine (HCQ), AZA, CsA, oral/intravenous CYC, RTX, and MMF, or splenectomy before danazol therapy. The mean daily dose of danazol during the 6-month observation period was 251.5 ± 78.0 mg. Nine patients (82.0%) showed a complete or partial response and GC-sparing effects throughout the series. Responses to danazol therapy for AIHA and ITP at 3 and 6 months were two (66.7%) and five (62.5%) and three (100%) and six (75.0%), respectively. Serial changes in hemoglobin (Hb) levels and platelet counts during the observation period are illustrated in Figure 1 and Figure 2. All the responsive patients were keeping the danazol therapy after the observation period. The duration of treatment in responsive patients was 38.6 ± 56.0 (range, 6 to 187) months. The major AEs were acne (*n* = 2, 18.2%) and the transient dose-related impairment of liver function (*n* = 1, 9.1%).

### 2.2. Literature Review

**Figure 3:**
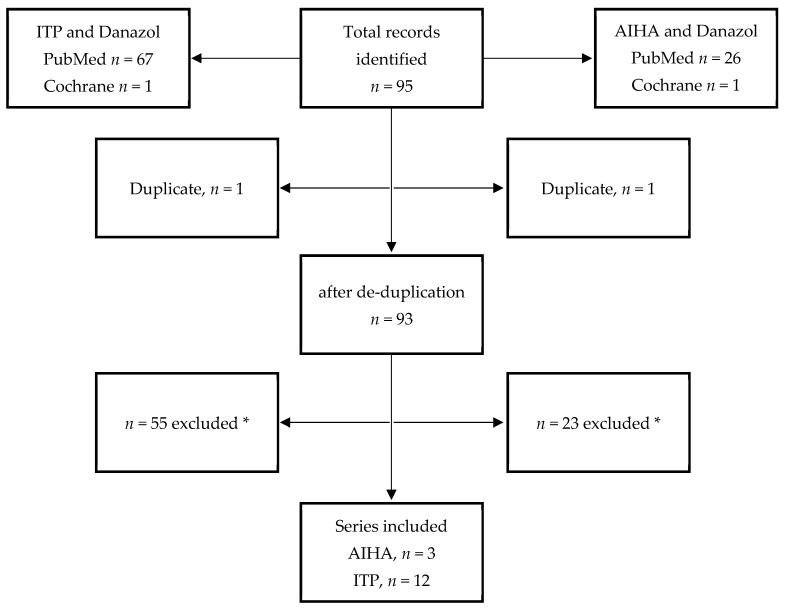
PRISMA flow diagram of study selection. ITP, immune thrombocytopenia; AIHA, autoimmune hemolytic anemia. * Excluded by title and reasons that do not pertain to study purposes.

In Step 2, 95 publications were retrieved, and full-text screening was performed; of these, 81 were excluded due to being duplicates and not meeting the inclusion criteria. Thus, 14 publications met our inclusion criteria for the final qualitative review (Figure 3). A list of the sorted series pertaining to AIHA or ITP and danazol therapy as well as a summary of the associated findings are illustrated in Table 2.

A total of 32 patients were included in three series of AIHA and danazol therapy. Most of the patients (*n* = 22, 68.8%) were female, with a mean age of 53.7 years. All the enrolled patients received GC treatment, and eight (25%) underwent splenectomy before the initiation of danazol. Of the 32 patients in these three series, 24 (75.0%) achieved a treatment response, including partial or complete, with an initial daily dose of danazol 400–800 mg, but no response rate was reported in those who received a low daily dose (<400 mg) of danazol. The duration of the disease available in the two series was 0.4 and 4.1 years each. AEs were reported in 7 of the 29 patients (24.1%) of the two series, including 3 patients (10.3%) with mild impairment of liver function without clinical symptoms, 1 (3.4%) with cramps and myalgia, 1 (3.4%) with facial hair growth, 1 (3.4%) with nervousness, and 1 (3.4%) with hair loss, but no patients withdrew from the therapy.

A total of 666 patients were included in the 12 series of ITP and danazol therapy. Similar to the AIHA series, 8 out of the 12 series reported that the patients were predominantly female (57.1–80%), with an available mean age range of 32–64 years in 7 series. The duration of the disease was available in four series and ranged from 2 to 6.3 years. All the included patients received various medications, including GC, AZA, CYC, intravenous immunoglobulin, and splenectomy, before the initiation of danazol therapy. The initial daily dose of danazol was 400–800 mg in nine (75%) series; a variable initial daily dose including >400 mg, 100–400 mg, and <100 mg in two (16.7%) series; and a daily dose of <400 mg in one (8.3%) series. The overall response was observed in 202 (59.6%) and 227 (68.0%) patients who received a high daily dose (400–800 mg) (*n* = 332) and a low daily dose (<400 mg) (*n* = 334), respectively.

As far as the available data, the response time to danazol was 1 week to 29 months in seven series, and the treatment duration of danazol was from 1.8 to 84 months in nine series.

Nine of the twelve series reported AEs. Ahn et al. reported major AEs, including weight gain (17%), lethargy (15%), and myalgia (14%), which did not lead to the discontinuation of therapy [20]. Maloisel et al. reported severe AEs in 9 of 57 patients (16%), including impaired liver function (*n* = 5, 55.6%), intracranial hypertension (*n* = 2, 22.2%), generalized skin rash (*n* = 1, 11.1%), and rhabdomyolysis (*n* = 1, 11.1%), in those who received a high initial daily dose of danazol (600 mg/day), which resulted in the discontinuation of therapy [27].

In terms of liver function impairment, 7 of the 12 series mentioned danazol-related AEs. A total of 573 participants were included in the seven series. We defined those who had impaired liver function but did not stop danazol therapy as having a mild form, whereas those who stopped it were considered to have a severe form. The proportion of patients with mild and severe forms of liver function impairment was 101 (17.6%) and 6 (1.1%), respectively. Meanwhile, the rate of mild and severe forms of liver function impairment in those who (*n* = 254) received a daily dose of danazol (400–800 mg) was 65 (25.6%) and 6 (2.4%), respectively. In the remaining patients (*n* = 319) who received a low daily dose of danazol (<400 mg), although 36 patients (11.1%) developed the mild form, the severe form was not noted.

## 3. Discussion

Danazol, an androgenic derivative of synthetic ethinyl testosterone, was first available in the 1960s and has been used to treat endometriosis since the 1970s. Since 1985, danazol has been used in the management of ITP and AIHA [17,20]. Although several hypotheses have been postulated, the mechanisms underlying the immune modulation of danazol are under investigation but remain obscure. It appears to be an effective immune modulator by increasing T-helper lymphocytes [31] and influencing Fcγ receptors in monocytes, decreasing platelet destruction [32]. In an in vitro study, Ahn et al. illustrated that danazol could protect erythrocytes from hypotonic osmotic lysis at low concentrations and proposed that danazol is incorporated into erythrocyte lipid layers in a reversible manner, expanding their surface area to be resistant to osmotic lysis. Additionally, danazol increased formed extra folds on erythrocyte membranes, and decreased erythrocyte osmotic fragility was noted one month after danazol therapy for ITP patients [33]. Horstman et al. indicated that danazol could protect opsonized platelets against complement-mediated lysis [34].

In the current series, the mean age was 52.6 ± 10.2 years, which was comparable with the previous series involving AIHA and ITP. Although aging was negatively correlated with the treatment outcome of danazol therapy in AIHA and ITP patients [17,20,27], the opposite finding was reported by Liu et al. [29], while we found aging was not related to treatment response in the current series.

Furthermore, the female predominance was the same as that reported in other studies [35,36]. The leading concomitant disease was SLE (81.8%) in our series, while its occurrence was much lower in the other series, except in the study by Arnal et al. They demonstrated the promising outcome of combination therapy with danazol and prednisone in those who had SLE with severe ITP and the possibility of withdrawing or to taper prednisone [24]. In addition, a review article also suggested that danazol is a helpful drug in the treatment of SLE patients, especially in those with refractory thrombocytopenia, autoimmune hemolytic anemia, and premenstrual flares [37]. However, whether SLE with AIHA or ITP is more responsive to danazol therapy needs further investigation.

The observation period of the response was 6 months in our series; however, as most of the previous series were retrospective in character, it was not inconsistent with the other series. Therefore, it is difficult to compare the onset and extent of the effect of danazol between the current series and the previous investigations. Long-term outcomes were interpreted in two series. One of the series indicated that a longer duration of danazol therapy was associated with a lower relapse rate [27]. Another series illustrated that patients who received continuous therapy had lower relapse rates than those who discontinued the therapy [29].

Meanwhile, in our series and other series, all the patients received GC treatment and at least two previous immunosuppressant regimens and were refractory or intolerant to the therapies adopted. We showed that the patients with AIHA and ITP responded favorably to a lower daily dose of danazol. All the responsive patients in our series had stationary Hb and platelet levels at a reduced dose of GCs after the initiation of danazol therapy, which was consistent with a previous report [19]. In addition, Ahn et al. reported the synergistic effect of GCs with danazol on ITP [20], and Feng et al. also disclosed that the monotherapy of danazol in ITP had a lower response rate [19]. All the danazol-responsive patients in the current series received reduced daily prednisolone (2.5–5 mg, or equivalent) after the observation period. Our investigation also confirms the experience of the previous series that danazol is an alternative for patients with AIHA or ITP who are refractory to conventional therapy. In addition, the response rate of the current AIHA series (100%) was higher than that of other AIHA series (70.6–100%), and the response rate of the current ITP series (75%) was also higher than that of 10 of the 12 series reviewed.

Regarding AEs, one patient experienced transient mild liver function impairment, and two patients experienced a sustained AE of acne, one of whom discontinued the therapy. The overall rate (*n* = 1, 33.3%) of AEs in AIHA in the current series was higher than that of AIHA in the other series (24.1%), and the overall rate of AEs in the ITP of the current series (*n* = 2, 18.1%) was lower than that of the ITP (31.7%) in the other series. In terms of liver function impairment, 1 patient in the current series (9.1%) had this AE and did not withdraw from the therapy, while 107 out of 573 participants (18.7%) in the other series had this AE, 6 of whom (1%) discontinued the therapy.

Evidently, the treatment response and AEs of the current series differed from those of the other series; this could be explained by several reasons. First, the daily dose of danazol in the previous series was 400–800 mg, while it was 200–400 mg in the current series. As is well known, the AE of danazol is dose-dependent [38]. A higher dose of danazol suggests a higher risk of AEs, especially liver function impairment. Second, as mentioned and presented in Figure 1 and Figure 2, the response rate of the current series at 6 months was higher than at 3 months. This finding suggests that danazol therapy for AIHA and ITP has a delayed effect. A longer observation period is needed to determine the full therapeutic effect of danazol on AIHA and ITP, as the observation period was not fully described in the other series. Third, SLE was the leading concomitant disease in our series but not in other series, and it is unknown whether SLE-related AIHA or ITP is more responsive to danazol therapy, which requires further investigation.

The experience of the current series not only confirms the effect of danazol on the management of AIHA and ITP but also suggests that a lower initial daily dose or maintenance dose (200–400 mg) of danazol can have a similar effect to 400–800 mg but with a lower AE of liver function impairment, which can hinder the discontinuation of therapy. Compared with the other series, the patients in the current series had a relatively longer duration of the disease. This suggests that danazol is effective not only in the acute-stage AIHA or ITP but also in the chronic stages. Danazol had a GC-sparing effect not only in our series but also in other series. This implies that danazol therapy for AIHA or ITP should be used in earlier stages rather than at a later stage or in refractory cases to avoid the potential side effects of GCs, immunosuppressants, and other costly biologics [39]. However, no available biomarker was useful in danazol therapy except complete blood count to observe the curative effect. Finally, due to the lag effect of danazol, a 6-month observation period is mandatory to determine the full effect of danazol on AIHA or ITP. In particular, the major AE of danazol therapy was the impairment of liver function and was the most common cause of withdrawal from the therapy in other series. It can occur at any stage of treatment. Hence, a regular follow-up of liver function throughout the treatment course is mandatory.

Our study has some limitations. The first drawback of our series was that a relatively small number of heterogeneous patients were enrolled, which hindered the chance of observing other AEs. The second drawback was that the current investigation was a real-world, retrospective, observational study, and no standard protocol of danazol therapy was used; thus, an observation or channel bias could not be avoided. The third drawback was that most of the sorted articles included small case series or personal experiences, which could not offer enough information to be compared with our series. Despite these limitations, the successful experience of current and past series in using danazol to treat AIHA or ITP suggests that danazol therapy is a favorable option to treat AIHA or ITP, but it needs further research.

In summary, a relatively lower initial daily and maintenance dose of danazol is a favorable alternative for treating refractory AIHA or ITP with fewer hepatic AEs and has a GC-sparing effect. The long-term effects of danazol require further prospective and large-sample investigations.

## 4. Materials and Methods

### Study Design and Ethical Approval

This study was carried out in two steps. In Step 1, participants with AIHA or ITP were included in the current study. The clinical characteristics of the participants, treatment response, and the AEs associated with danazol therapy are presented. In Step 2, a literature review on AIHA or ITP with danazol therapy was carried out by searching the Medline (PubMed) and Cochrane Library of reports published from 1984 to January 2022. This study was approved (code, 202201221B0) by the Institutional Review Board of the Chang Gung Medical Foundation in Taoyuan, Taiwan.

Step 1: sample

We recruited consecutive patients with AIHA or ITP who had been refractory or intolerant to GCs, immunosuppressants, or splenectomy and received danazol therapy between 2008 and June 2022 in the Department of Rheumatology at Chang Gung Memorial Hospital, Kaohsiung. The diagnosis of AIHA was based on a positive direct antiglobulin test, high serum lactate dehydrogenase level, reticulocytosis, spherocytosis in peripheral blood smears, and the exclusion of other causes of hemolysis. ITP was diagnosed based on the presence of thrombocytopenia with a platelet count of <100 × 10^3^/μL and the exclusion of other etiologies of thrombocytopenia. A bone marrow study had been performed for all ITP patients before danazol therapy, but only two biopsies were performed in this institution with the available reports. One pathological report of a patient with SLE and pancytopenia showed severe megakaryocytic hypoplasia, consisting of aplastic anemia, and the other report of a patient with ITP disclosed essentially normal marrow, with no increase in plasma cells, considering the monoclonal gammopathy of undetermined significance. Both reports were absent of karyotype analysis.

The treatment response to danazol in our cohort was recorded at 3 and 6 months after the initiation of therapy. The treatment response for patients with AIHA was categorized as complete, partial, or no response. A complete response was defined as a steady Hb level ≥ 10 g/dL with a daily GC dose of <5 mg of prednisolone (or equivalent). A partial response was defined as the achievement of steady Hb levels when the daily dose of prednisolone (or equivalent) was 5–10 mg. No response to therapy was assumed when a sustained Hb level ≥ 10 g/dL was not achieved at any of the daily doses of prednisolone (or equivalent).

The treatment response for patients with ITP was based on the modified criteria proposed by the American Society of Hematology [40]. The response was categorized as complete, partial, and no response according to a platelet count of ≥100 ×10^3^/μL; a double of the baseline, 30 × 10^3^/μL but <100 × 10^3^/μL; and any platelet count less than 30 × 10^3^/μL or less than double the baseline count, respectively, 6 months after the initiation of danazol therapy with a concomitant daily prednisolone dose of ≤10 mg.

Step 2: literature review and case series

The articles on patients with AIHA or ITP treated with danazol therapy published from 1984 to January 2022 in any language were systematically searched with the terms combined with the Boolean operators AND/OR. The key terms used for the search in the PubMed and Cochrane Library were “immune thrombocytopenia,” “autoimmune hemolytic anemia,” and “danazol”. The search items were translated into multiple matching synonyms to broaden the results. A systematic review in accordance with PRISMA guidelines was conducted. The case reports not related to AIHA or ITP, duplicate studies, and articles without abstracts were excluded (Figure 3).

## Figures and Tables

**Table 1:** The demographics, clinical characteristics, and treatment response of the current series.

Patients	Disease	Sex	Age	Concomitant Diseases	Disease Duration (Year)	Previous Therapy	Danazol Daily Dose		Response	Adverse Events
							Initial	Mean (6 mo)		
1	AIHA	F	41	SLE	4	GC, AZA	200	167	Complete	Acne
2	AIHA	F	60	Chronic ischemic heart disease, SLE	20	GC, AZA, MMF	400	200	Partial	-
3	AIHA	F	48	SLE	6	GC, AZA, CsA, MMF, CYC (IV/Oral),	200	200	Partial	-
4	ITP	F	43	Hepatitis b, liver cirrhosis, SLE	14	GC, AZA, CYC (oral),	200	200	Complete	-
5	ITP	F	49	SLE, aplastic anemia	11	GC, AZA, CsA,	400	233.3	Partial	-
6	ITP	F	63	DM, CKD, SLE	17	GC, AZA	200	200	Complete	-
7	ITP	F	43	SLE	5	GC, CsA CYC (IV/Oral)	400	233.3	Complete	Liver Function impairment
8	ITP	F	74	MGUS, Hypothyroidism, DM	21	GC, RTX, CYC (Oral)	400	400	Partial	-
9	ITP	F	50	-	4	GC, AZA, MMF, CYC (IV), RTX, splenectomy	400	333.3	Complete	-
10	ITP	F	59	Hyperthyroidism, SLE	31	GC, AZA, CsA	400	- *	No	Acne
11	ITP	F	49	SLE	15	GC, CsA	400	366.7	No	-

AIHA, autoimmune hemolytic anemia; AZA, azathioprine; CKD, chronic kidney disease; CsA, cyclosporine; CYC, cyclophosphamide; DM, diabetes mellitus; GC, glucocorticoid; HCQ, hydroxychloroquine; ITP, immune thrombocytopenia; MGUS, monoclonal gammopathy of undetermined significance; MMF, mycophenolate mofetil; RTX, rituximab; SLE, systemic lupus erythematosus. * Withdrawn due to adverse events in month 1.

**Table 2:** Published studies on danazol for the treatment of immune thrombocytopenia and autoimmune hemolytic anemia.

Author	Diseases	Cases	Female (%)	Age	Disease Duration (Year)	Previous Therapies	Initial Danazol Dose/Day	Response, %
Ahn 1985 [17]	AIHA	12	91.7	52.2	4.1	GC, CYC, AZA, splenectomy	600–800	75.0
Manoharan 1987 [18]	AIHA	3	66.7	63.3	0.4	GC, splenectomy	600	100
Pignon 1993 [19]	AIHA	17	58.8	53.1	-	GC, splenectomy, immunosuppressants	400–600	70.6
Current series	AIHA	3	100	49.7	10	GC, AZA, CsA, MMF, CYC	200–400	100
Manoharan 1987 [18]	ITP	5	80	55.8	2.7	GC, splenectomy, CYC, VCR, AZA	600	80.0
Ahn 1989 [20]	ITP	96	62.5	52	-	GC, splenectomy	400–800	61.4
Edelmann 1990 [21]	ITP	7	57.1	64	6.3	GC, AZA VCR, colchicine	800	57.1
Kondo 1992 [22]	ITP	14	78.6	54	-	GC, AZA, IVIG, splenectomy	100–400	78.6
Schiavotto 1993 [23]	ITP	17	-	-	-	-	400–800	56.0
Arnal 2002 [24]	SLE + ITP	18	-	-	2.2	GC, AZA, CYC, HCQ, IVIG,	50–600	50.0
Andrès E 2003 [25]	ITP	33	60	-	-	GC, splenectomy	600	72.0
Zimmer 2004 [26]	ITP	37	-	-		-	600	73.0
Maloisel 2004 [27]	ITP	57	63	54	2	GC, IVIG, splenectomy	600	67.0
Daou 2008 [28]	ITP	15	-	-	-	GC, splenectomy	400	60.0
Liu W 2016 [29]	ITP	319	69.9	51	-	GC	100–300	65.0
Feng 2017 [30]	ITP	48	60.4	32	-	GC, AZA, CsA, MMF, IVIG, RTX, VCR, rHuTPO	400	43.8
Current series	ITP	8	100	53.8	14.8	GC, AZA, CsA, MMF, CYC, RTX, splenectomy	200–400	75.0

AIHA, autoimmune hemolytic anemia; AZA, azathioprine; CsA, cyclosporine; CYC, cyclophosphamide; GC, glucocorticoid; HCQ, hydroxychloroquine; ITP, immune thrombocytopenia; IVIG, intravenous immunoglobulin; MMF, mycophenolate mofetil; rHuTPO, recombinant human thrombopoietin; RTX, rituximab; SLE, systemic lupus erythematosus; VCR, vincristine.

## Data Availability

Not applicable.
